# Analyzing heavy metal contamination for one of the high-rate consumption fruits in Iran: A probabilistic health risk assessment

**DOI:** 10.1016/j.heliyon.2024.e30392

**Published:** 2024-04-28

**Authors:** Mohammad Rezvani Ghalhari, Nayereh Rezaei Rahimi, Mohammad Fahiminia, Elahe Noruzzade, Abooalfazl Azhdarpoor, Zeynab Koochakzadeh, Habib Vakili, Reza Fouladi-Fard

**Affiliations:** a Department of Environmental Health Engineering, School of Public Health, Tehran University of Medical Sciences, Tehran, Iran; b Student's Scientific Research Center, Tehran University of Medical Sciences, Tehran, Iran; c Department of Environmental Health Engineering, School of Health, Shiraz University of Medical Sciences, Shiraz, Iran; d Research Center for Environmental Pollutants, Department of Environmental Health Engineering, Faculty of Health, Qom University of Medical Sciences, Qom, Iran; e Department of Health, Safety and Environment, Pasteur Institute of Iran, Tehran, Iran; f Environmental Health Research Center, School of Health and Nutrition, Lorestan University of Medical Sciences, Khorramabad, Iran

**Keywords:** Cucumber (Cucumis sativus L.), Heavy metals, Risk assessment, Fruits, ICP-OES

## Abstract

Good health and well-being is one of the sustainable development goals (SDGs) that can be achieved through fruit consumption. This study measured cucumber (Cucumis sativus L.) heavy metal concentrations. Inductively coupled plasma-mass spectrometry (ICP-OES) was used to analyze the samples for heavy metal content. The uncertainty and sensitivity analyses of carcinogenic and non-carcinogenic heavy metal intake via cucumber (*Cucumis sativus L.*) consumption were assessed by Monte Carlo simulation. The mean ± SD levels of Cu, Pb, Zn, Cd, and As were determined to be 157.87 ± 128.54, 33.81 ± 6.27, 288.46 ± 114.59, 35.22 ± 18.67, and 33.6 ± 18.1 μg/kg, respectively. The 95th percentile of HI related to heavy metal intake via cucumber (*Cucumis sativus L.*) among children and adults were 2.64 and 1.75, respectively. Also, the 95th percentile of ELCR related to heavy metal were 8.26E-4 and 4.14E-3 among children and adults, respectively. The 95th percentile of LTCR of As among adults and As, Cd, and Pb among children were in the WHO target range (1E-04 to 1E-06) so reducing the concentration of them can help to reduce overall LTCR. When HQ and LTCR are below the cut limits, reducing heavy metals in high-consumption meals is a good way to lower them. In general, due to the wide consumption of various fruits, such as cucumber (*Cucumis sativus L.*), the concentration of environmental pollutants in their edible tissues should be monitored regularly, and the concentration of pollutants in these tissues should be minimized by proper planning.

## 1 Introduction

Fruits serve a vital role in the human diet due to their provision of critical nutrients, including vitamins, carbohydrates, and dietary fiber [1]. It's common knowledge that certain meals may shield humans from illness [2]. Cucumbers (*Cucumis sativus L.*) are one of the fruits that exhibits substantial appeal and is widely consumed worldwide [3]. Specially, Cucumbers (*Cucumis sativus L.*) are widely consumed in the Middle East due to their nutritious value, delicious taste, and high water content [4]. Cucumber (Cucumis sativus L.) is an ingredient in Iranian salads, which are one of the most widely eaten desserts [5]. Several studies showed that pollutants such as microplastics, polycyclic aromatic hydrocarbons, heavy metals, antibiotics, etc. have the ability to accumulate in the edible parts of cucumber (*Cucumis sativus L.)* [[6], [7], [8], [9], [10]].

Anthropogenic activities and industrial expansion, including mining, textile production, smelting operations, the dye and steel industries, and chemical manufacture, have the potential to discharge heavy metals into the environment [11,12]. In recent decades, the presence of heavy metals due to their properties in soils has emerged as a significant concern for food safety [13]. The origins of heavy metals in soils may be traced back to both human activities and the historical geological processes that have influenced their presence [14,15]. So, it's unsurprising that heavy metals in fruit may enter the human body by ingestion, build up in the organs through the food chain, and treat human health [16,17]. Since the human body is unable to eliminate heavy metals, reducing exposure to heavy metals is necessary because even less exposure may impair organ function [18].

The biochemical properties of heavy metals can be changed after entering human organs [19]. Liver, cardiovascular, renal, gastrointestinal, pulmonary, intestinal, and skeletal damage may result from heavy metal accumulation over the safe threshold. In addition, heavy metals have been linked to cognitive impairment, neurological disease, and malignancy [[20], [21], [22], [23]]. Vegetable and fruit heavy metals were commonly observed globally, with As, Cd, Cr, and Pb routinely surpassing Food and Agriculture Organization (FAO) and World Health Organization (WHO) acceptable thresholds [24,25].

Contaminated water and soil can increase the concentration of heavy metals in plant tissues such as Cucumbers (*Cucumis sativus L.*) [26,27]. So, cucumbers (*Cucumis sativus L.*) can contain a wide range of heavy metals based on their irrigation water source [28]. Salads are the main type of dessert, and because cucumber (*Cucumis sativus L.*) is a main ingredient in salads in many parts of the world, especially in Iran, reducing the contaminants in salad ingredients can increase their nutritional value and positive effects. Therefore, the aims of the present study were to: 1) determine heavy metal levels in cucumber (*Cucumis sativus L.)* collected from the markets in Qom in the central region of Iran; and 2) evaluate the carcinogenic and non-carcinogenic risks attributed to heavy metal residue in cucumber (*Cucumis sativus L.*) among children and adults.

## 2 Material and methods

### 2.1 Reagents

All of the reagents used were of analytical grade. Ultrapure water (resistivity 18.2 M/cm) acquired from a Direct-Q3 water purification system (Millipore Corporation, Burlington, MA, USA) was used to produce the solutions. Fisher Scientific (Waltham, MA, USA) supplied trace metal grade concentrated nitric acid (67–70 %) and concentrated hydrochloric acid (34–37 %). The HNO3 was diluted in ultrapure water to generate a washing solution that was 50 % (v/v) and 2 % (v/v) for standard dilution and cucumber (*Cucumis sativus L.*) samples acidification, respectively. Sigma Aldrich (St Louis, MO, USA) provided 30 % hydrogen peroxide. Prior to conducting inductively coupled plasma atomic emission spectrometry (ICP-OES) analysis, the materials underwent filtration using syringe filters with cellulose acetate membranes, specifically the 0.45 μm VWR (Radnor, PA, USA) syringe filters.

### 2.2 Area of study

The cucumber (*Cucumis sativus L.*) samples were obtained from the markets in Qom, a city situated in central Iran at coordinates 44°34′37″ N and 55°33′27″ E. Qom is located around 130 km distant from Tehran and is situated at a height of 936 m above the mean sea level. According to the data from the 2016 census, the population of the area under question amounts to 1.2 million inhabitants. Based on meteorological data, the mean relative humidity was around 41 %, the mean precipitation is 148.2 mm/yaer, and the mean temperature is 18.2 °C annually. As a result of these agricultural activities, it is approximated that a substantial quantity of around 14,000 tons of crops are yielded on an annual basis [[29], [30], [31], [32], [33]].

### 2.3 Sample collection

**Fig. 1:**
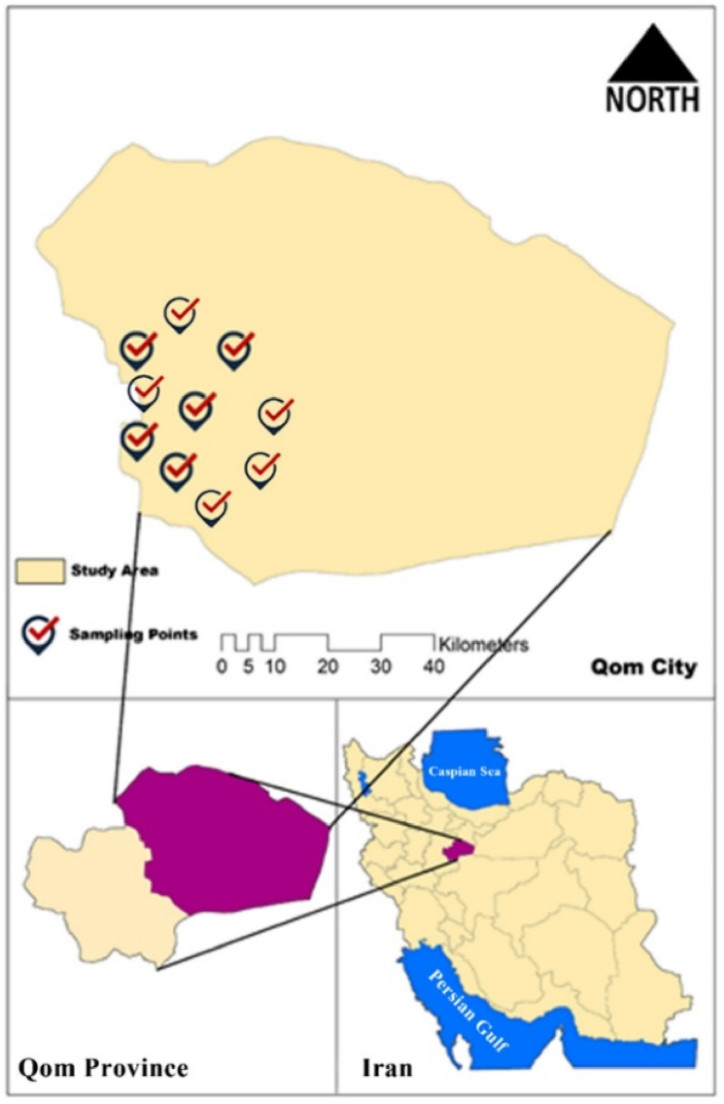
The location of study area and the cucumber (*Cucumis sativus L.*) sampling point.

To carry out this study, cucumber (*Cucumis sativus L.*) that trade in 10 large stores (see Fig. 1) of the Qom were collected. 30 samples were taken, one each from three different stores' collections of cucumbers (Cucumis sativus L.). Polyethylene bags were used to carry the samples from the markets to the lab, then were stored in a sterile glass container at 4 °C until the day of preparation and analysis. Studies involving plants, whether conducted in experimental settings or natural environments, adhere to pertinent institutional, national, and international regulations and protocols for collecting plant samples [34].

### 2.4 Sample preparation

In the first stage of cucumber (*Cucumis sativus L.*) preparation, 200 g of collected sample were weighted and dried in an oven at 105 °C for 4 h. Then the dried samples were crushed with a microhammer (Wiley Mill, Thomas Scientific) to have uniform sizes of 2 mm, which were determined by sieving. However, in many countries, the skin of a cucumber is an inedible part, but in Iran and many countries in the Middle East, cucumber skin is considered one of the edible parts, and according to the existing Islamic culture, in order to prevent wastage and produce less food waste in these countries, cucumber skin is also one of the edible parts. So, in the present study, to achieve the actual concentration to which consumers have been exposed, the cucumber peel has been crushed. To prevent any potential contamination between samples, the microhammer and sieve underwent a meticulous cleaning process using triple-distilled water and were subsequently dried after each use. The samples that were made ready for analysis were placed in uncontaminated, hermetically sealed polyethylene containers. Then, 2 g of the powdered sample was weighted and transferred to a sterilized crucible. The crucible was put on a hot plate to remove the moisture from the powdered samples, which were named pre-ash. These pre-ash samples transfer to a muffle furnace, which is adjusted at 480 °C for 2 h. In the next stage, the ash samples were digested using a 10 % nitric acid (HNO_3_) solution, and then the digested samples were filtered by a 0.45 μm filter into 25 ml polyethylene volumetric flasks. Subsequently, the filtered solutions that were prepared to inject into ICP-OES should be diluted using a 1 % (v/v) nitric acid (HNO_3_) solution [35,36].

### 2.5 Instrumental analysis

The ICP-OES (SPECTRO, Germany) was applied to analyze the concentration of heavy metals in cucumber (*Cucumis sativus L.)* samples. The ICP-OES's operating conditions are presented in Table 1. The calibration curves consisted of a minimum of six data points, exhibiting linear best-fit correlation coefficients exceeding 0.995. The quantification of heavy metal concentrations was conducted in μg/Kg, and subsequently converted to μg/g DW using Eq. (1):

(Eq.1) $$\frac{\mu g}{g}\;dw=\frac{\frac{ppb}{100}\times 1000}{0.002}$$

**Table 1:** ICP-OES condition for detection of heavy metals in cucumber (*Cucumis sativus L.*).

Parameter	Value and Note	Unit
Torch type	Flared	
EOP Torch	2.5	mm
Type of detector Solid state	CCD	
Type of spray chamber Cyclonic	Modified Lichte	
RF generator frequency	27.12	MHz
Auxiliary gas flow rate (L/min)	0.9	(L/min)
Nebulizer gas flow rate (L/min)	0.85	(L/min)
Plasma, auxiliary and nebulizer gas	Argon	
Plasma gas flow rate	14.5	(L/min)
Pump type	four-channel	
Measurement replicates	3	
Sample uptake time	330	S
Sample injection pump rate	30	RPM
pre-wash pump Rate	60	RPM
Rinse time off	45	S

### 2.6 Method validation, LODs, and LOQs

Table 2 displays the values for the limit of detection (LOD) and the limit of quantification (LOQ) of the ICP-OES technique. These values were employed in the analysis of heavy metal concentrations in cucumber (Cucumis sativus L.) samples.

**Table 2:** The LOD, LOQ value and recovery % of heavy metal that detected by ICP-OES.

Heavy Metals	LOD (μg/Kg)	LOQ (μg/Kg)	R (%)
Cd	0.6125	1.8375	99.982
Pb	27.075	81.225	99.987
As	12.5	37.5	99.994
Cu	3.825	11.475	99.962
Zn	3.375	10.125	99.996

The stock-standard solutions were used to carry out the calibration procedure. In this particular investigation, stock-standard solutions were prepared utilizing concentrations of 100, 500, and 1000 ppm. Following the necessary preparations, 10 mL of initial samples were supplemented with standard stock solutions. Subsequently, the resulting mixture was agitated for 1 min. To mitigate the influence of the matrix effect, agitated solutions were employed in the ICP-OES method [37]. The recovery of metal ions (R, %) was determined using Eq. (2):

(Eq.2) $$R\left(\%\right)=\frac{C_{0}-C_{e}}{C_{e}}\times 100$$

where С_0_ is described as the concentration of spiked analyte (μg/Kg) and Сe is the analyte concentration in the unspiked sample (μg/Kg).

### 2.7 Human health risk assessment

Generally, the health risk assessment involves four hierarchy steps: 1) hazard identification; 2) dose-response assessment, which can show the effect of increasing levels of heavy metal concentration on adverse effects; 3) exposure assessment is undertaken at a local and specific level, focusing on the particular location (which in the present study is central Iran); 4) risk characterization [38]. All mentioned steps are defined in detail in the Pirhadi et al. (2022) study [39].

Monte Carlo simulation (MCS) has gained significant popularity as a human health risk assessment approach that is applied to water and food pollution and contamination, respectively [40]. The Oracle Crystal Ball software (version 11.1.2.4, Oracle, Inc., USA) is an Excel ribbon that can be used to assess the health risk using the MCS approach, also this software can carried out the sensitivity analysis. The parameters that using for health risk assessmnent in MCS approach are obtained from animal experiments; so there is an uncertainity when this method used in human study which this uncertainity should be considered when MCS method use for study that related to human [41]. The MSC technique employed a conservative approach by considering the 95th percentile as the value for assessing the worst potential health risk [42].

### 2.8 Non-carcinogenic health risk assessment

The daily diet consists of several trace elements, including heavy metals, which have the potential to enhance both non-carcinogenic and carcinogenic impacts. In order to assess the impacts, it is necessary to determine the chronic daily intake (CDI) (mg/kg.d) of heavy metals from cucumber (*Cucumis sativus L.*) using the equation (**Eq. 3**) proposed by Chien et al. (2002) [43]:

(Eq.3) $$CDI=\frac{C\times IR\times \text{CF}\times EF\times ED}{BW\times AT}$$

The concentration of heavy metals in cucumber (*Cucumis sativus L.*) samples (μg/kg) is denoted by C, the ingestion rate (IR) represents the daily intake of cucumber (*Cucumis sativus L.*) among Qom consumers (Kg/d), the conversion factor (CF) was equal to 1E-03, the exposure frequency (EF) refers to the frequency of cucumber (*Cucumis sativus L.*) consumption in a year, the exposure duration denotes by ED, the average body weight (BW) of different age groups is used for human health risk assessment, and the averaging time (AT) for non-carcinogenic health effects is calculated as ED × 365, which varies among age groups; however, for assessing carcinogenic health effects, this parameter is equal to 25550 for all age groups. Table 3 displays the factors employed for evaluating the associated health risk posed by heavy metals in the cucumber (Cucumis sativus L.). The determination of non-carcinogenic health risk assessment involved the calculation of the hazard quotient (HQ) using Eq. (4)., as developed by the United States Environmental Protection Agency (US-EPA) [43].

(Eq.4) $$\text{HQ}=\frac{CDI}{RfD\;}$$

where, CDI calculated by Eq. (3), and the oral reference dose denotes by RfD (mg/kg/day) of heavy metals presented in Table 3.

**Table 3:** Effective parameters on health risk assessment related to heavy metals in cucumber (*Cucumis sativus L.*) among different age groups.

Parameters	Symbol	Unit	Distribution	Children	Adults	References
ingestion Rate	IR	Kg/d	Lognormal	0.232	0.345	[44]
heavy metals concentration	C	μg/kg	Lognormal	–	–	Table 4
Body Weight	BW	Kg	Normal	20	70	[45]
Average Time non-carcinogen	AT	Days	Uniform	2190	25550	[46]
Average Time carcinogen	AT	Days	Uniform	25550	25550	[46]
Exposure Frequency	EF	Days/Year	Uniform	365	365	[46]
Exposure Duration	ED	Year	Uniform	6	70	[46]
RfD Pb		mg/kg/day	–	0.0035		[47]
RfD Cu			–	0.04		
RfD Zn			–	0.3		
RfD As			–	0.003		
RfD Cd			–	0.001		

Heavy metals have cumulative properties, so these elements can accumulate in human tissues. Based on this property, the actual risk of heavy metal levels in cucumber (*Cucumis sativus L.*) is presented by the hazard index (HI), which is calculated by Eq. (5):

(Eq.5) $$\text{HI}=\sum_{i}^{n}HQ$$

The value of the HI serves as an indicator of the potential adverse effects associated with the concentrations of heavy metals in the cucumber (*Cucumis sativus L.*). When HI is greater than 1, it suggests that there is a likelihood of adverse effects resulting from the presence of heavy metals, and non-cancer risks are probable. Conversely, when HI is less than 1, it indicates that there is no evidence of adverse effects associated with the concentrations of heavy metals in the cucumber (Cucumis sativus L.), and the likelihood of non-carcinogenic risks occurring is low [48].

This research used lifetime cancer risk (LTCR) to figure out how dangerous it is for people to get cancer if different heavy metals are found in cucumbers (*Cucumis sativus L.*). The LTCR values were computed using Eq. (6). The calculation of the carcinogenic risk, known as the cancer risk due to CDI, was performed using Eq. (3). The cancer slope factor (CSF) was utilized to determine the particular risk associated with each heavy metal. For Pb, Cd, and As, the CSF values were determined to be 0.0085, 0.38, and 1.5 mg/kg/day, respectively Table 4.

(Eq.6) LTCR=CDI×CSF

**Table 4:** Descriptive statistics of heavy metals in cucumber (*Cucumis sativus L.*) samples and comparison with FAO/WHO standard.

Heavy metals	Mean (μg/Kg)	±SD (μg/Kg)	FAO/WHO (μg/Kg)	Exceeded limits (%)	*P*-Value (CI 95 %)	P95	var	CV
As	28.58	13.52	100	0	**0.001>**	6.44	287.33	0.537
Pb	33.81	6.27	500	0	**0.001>**	2.66	51.10	0.338
Cd	35.22	18.67	50	16.66	**0.001>**	8.75	529. 6	0.853
Cu	157.87	128.44	200	26.66	0.08	49.07	16648.11	0.824
Zn	288.46	114.59	2000	0	**0.001>**	42.79	13133.09	0.397

Based on cumulative heavy metals property, Excess Lifetime Cancer Risk (ELCR) should be calculated. So, the LTCR of each heavy metals in cucumber (*Cucumis sativus L.*) should be summed with other LTCRs based on Eq. (7):

(Eq.7) $$\text{ELCR}=\sum_{i}^{n}LTCR$$

The interpretation of the ELCR is as follows, taking into account a conservative scenario at the 95th percentile. If the ELCR value was below 1E-06, it indicated that there was no significant presence of heavy metals associated with cancer risk in the cucumber (Cucumis sativus L.). Consequently, additional investigation or prioritization was not deemed necessary. Additional research was deemed necessary prior to making any decisions or formulating a strategy for low-priority actions in two scenarios: (1) when the ELCR fell within the range of 1E-04 to 1E-06, which aligns with the World Health Organization's (WHO) target for carcinogenic risk assessment, and (2) when the ELCRs exceeded 1E-04. In both cases, further investigation was deemed essential before proceeding [49].

## 3 Results and discussion

### 3.1 The concentration of heavy metals in cucumber (*Cucumis sativus L.*)

The heavy metals concentrations in the cucumber (*Cucumis sativus L.*) samples that were purchased from the Qom vegetable market have been measured. The mean ± SD levels of heavy metals in the cucumber (*Cucumis sativus L.*) samples and other descriptive statistics of heavy metals concentration in samples are presented in Table 4, high concentrations of heavy metals in cucumber (*Cucumis sativus L.*) can be due to the use of soil or water that is contaminated with high concentrations of heavy metals through different ways [50]. The study of Mohsen et al. (2019) showed that the average levels of heavy metals in the sea cucumber were Pb (10.17–25.72 mg/kg), Cu (4.75–52.66 mg/kg), Zn (10.33–82.72 mg/kg), As (3.01–12.42 mg/kg), and Cd (0.04–0.17 mg/kg) [51], In both aquatic and terrestrial plants, the distribution of heavy metals in plant sections is typically unequal [52,53]. Organic matter content plays a crucial role in influencing the availability and mobility of HMs in soils. This is due to its ability to decrease the bioavailability of heavy metals in soils through processes such as adsorption or the formation of stable complexes with humic substances [54].

The findings from Table 4 indicate that in 16.66 % of the samples, the concentration of Cd exceeded the FAO/WHO standard [55], while the concentration of Cu exceeded the standard in 26.66 % of the samples. Additionally, the T-test results presented in Table 4 demonstrate a significant disparity between the concentrations of heavy metal levels in cucumber (Cucumis sativus L.) samples and the FAO/WHO standard (CI = 95 %, *P*-value <0.001). However, the variation in Cu levels compared to the FAO/WHO standard was found to be insignificant (CI = 95 %, *P*-value = 0.08). The concentration of heavy metals in different fruits in different regions is compared in Table 5.

**Table 5:** Comparison of the mean concentration of heavy metals in various fruits.

Fruit type	Country	As (μg/Kg)	Pb (μg/Kg)	Cd (μg/Kg)	Cu (μg/Kg)	Zn (μg/Kg)	Ref.
cucumber (Cucumis sativus L.)	Iran	28.58	33.81	35.22	157.87	288.46	Present study
cucumber (Cucumis sativus L.)	Egypt	–	145070	14120	92260	74140	[56]
cucumber	China	380	672	118	2440	78800	[57]
Mango	Bangladesh	13	642	5	7891	604	[58]
Banana	Bangladesh	–	3	–	946	235	[58]
Fresh fruits	China	57	11.3	3.3	741.2	1852.7	[59]

### 3.2 Human health risk assessment

The risk assessment should be conducted with a conservative approach, taking appropriate actions based on the assessment. In numerous risk assessment studies, the 95th percentile is commonly adopted as a conservative threshold. Variations exist among human and animal characteristics, as well as within human populations residing in the same community, necessitating the inclusion of uncertainty analysis. Furthermore, there are several research gaps that can be addressed through uncertainty analysis [60].

#### 3.2.1 Non-carcinoenic effect

**Fig. 2:**
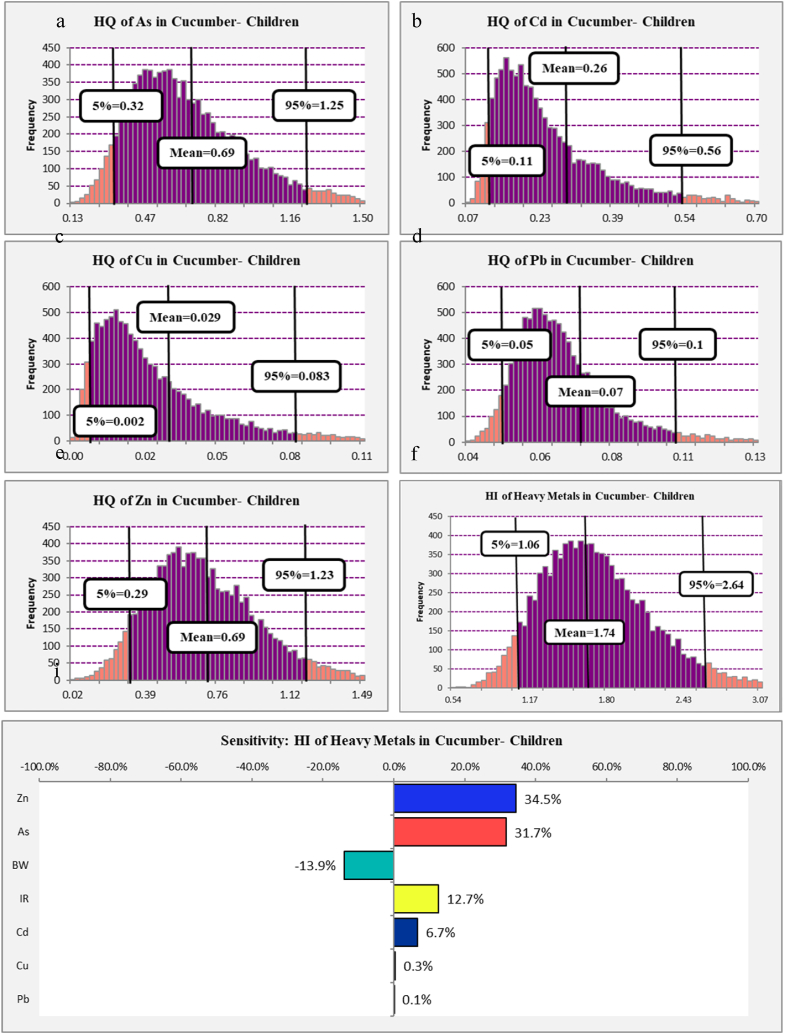
Histograms of the simulated HQ (a–e), HI (f) and sensitivity analysis (i) of heavy metals in cucumber (*Cucumis sativus L.*) among children.

The estimated HQ of heavy metals among children that consume the cucumber (*Cucumis sativus L.*) presented in Fig. 2. Results shows that the 95th percentile of HQ of As and Zn intake via cucumber (Cucumis sativus L.) consumption among children was 1.25 and 1.23, respectively, which were more than 1 (HQ > 1), so concentrations of As and Zn in the cucumber (*Cucumis sativus L.*) have some adverse effect for children. Also, results presented that the 95th percentile of simulated HQs of Cd, Pb, and Cu among children via cucumber (*Cucumis sativus L.*) consumption were 0.56, 0.1, and 0.08, respectively. As you can see, the 95th percentile of HQs was lower than 1 (HQ < 1), so the level of Cd, Pb, and Cu in cucumber (*Cucumis sativus L.*) has no adverse effect in the long term for children [61], and the concentration of these heavy metals (Cd, Pb, and Cu) in cucumber (*Cucumis sativus L.*) is in a safe range.

Decreasing cucumber (*Cucumis sativus L.*) intake for children is helpful to reduce estimated HQs, although it cannot be a definitive and logical solution, so environmental monitoring and taking appropriate action to reduce the concentration of heavy metals in water and soil used for irrigation and agriculture are necessary [62]. According to Eq. (2) another action that can reduce the risk is increasing BW [63], but it should be noted that increasing BW itself is one of the risk factors that can lead to many other diseases [64,65], so increasing BW cannot be used as a logical and correct solution. But it should be noted that in order to reduce the risk, the body mass index (BMI) of children should be normal and acceptable [66], because if children are underweight due to the reduction of the denominator of Eq. (2), the calculated risk will be very high, which can threaten children's health.

Heavy metals have an accumulation property in the human body tissues, therefore, the entry of various types of these metals into the human body and accumulation in the body tissues can cause an increase in HQ with their synergistic effect [67]. As shown in Fig. 2 f the HI that related to heavy metals intake via cucumber (*Cucumis sativus L.*) consumption among children was calculated to presents the synergistic effect; the results shown that 95th percentile of HI was 2.64 which is more than 1, The HI increasing can be dangerous regarding the potential health ramifications linked to the exposure of heavy metals. Nevertheless, it is crucial to acknowledge that a HI exceeding 1 does not inherently indicate the occurrence of detrimental health consequences. The assessment suggests that additional inquiry and the implementation of risk management methods are required in order to minimize potential risks [68].

The sensitivity analysis of calculated HI can help to determine the effect of each parameter that can increase or decrease the HI. The sensitivity analysis of calculated HI of heavy metals in cucumber (*Cucumis sativus L.)* among children is presented in Fig. 2 i. The results show that Zn, As, IR, Cd, Cu, and Pb have 34.5 %, 31.7 %, 12.7 %, 6.7 %, 0.3 %, and 0.1 % positive effects on increasing HI among children. Also, BW has a 13.9 % decreasing effect on HI among children. So, decreasing the concentration of heavy metals such as As and Zn can be considered the best action to decrease the HI [69] because As and Zn together have about a 65 % positive effect on the HI.

**Fig. 3:**
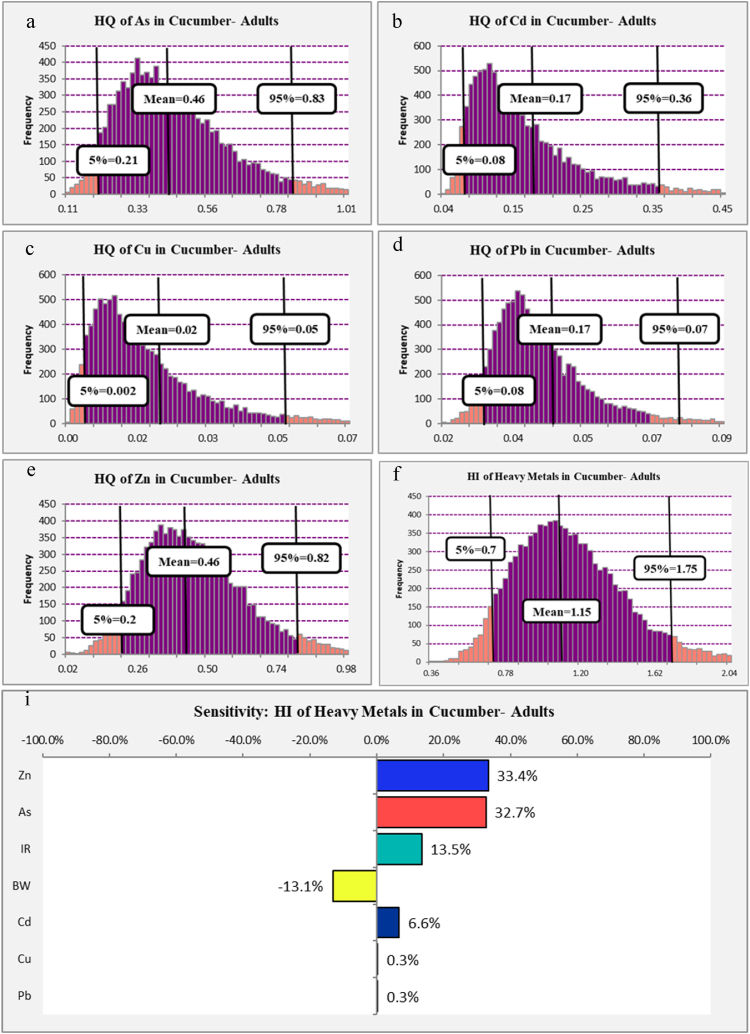
Histograms of the simulated HQ (a–e), HI (f) and sensitivity analysis (i) of heavy metals in cucumber (*Cucumis sativus L.*) among adults.

The estimated HQ of target heavy metals in the cucumber (*Cucumis sativus L.*) among adults presented in Fig. 3 shows that the 95th percentile of HQs in all heavy metals intake via cucumber (*Cucumis sativus L.*) consumption among adults were lower than 1 (HQ < 1). The simulated HQ for As, Zn, Cd, Pb, and Cu were 0.83, 0.82, 0.36, 0.07, and 0.05, respectively. According to these results and the principles of risk assessment, no special action is needed to prevent the occurrence of diseases related to heavy metals among adults that using cucumber (*Cucumis sativus L.*). However, sensitive groups should always be taken into account, and as much as possible, food items that have the lowest amount of heavy metals should be used. As a result, reducing the concentration of heavy metals in high-consumption foods should be considered a principle to reduce HQ, even in cases where the value of HQ is less than 1.

According to accumulation property of heavy metals in the human body tissues, the synergistic effect of heavy metals can threaten human health, so to consider synergistic effect of heavy metals the HI was calculated. As shown in Fig. 3 f the 95th percentile of HI that related to heavy metals intake via cucumber (*Cucumis sativus L.*) consumption among adults was 1.75 which is more than 1 (HI > 1), which can be dangerous for long term and can cause of the chronic disease.

The sensitivity analysis of the calculated HI can help determine the effect of each parameter that can increase or decrease the HI. The sensitivity analysis of the calculated HI of heavy metals in cucumber (*Cucumis sativus L.*) among adults is presented in Fig. 3i. The results show that Zn, As, IR, Cd, Cu, and Pb have 33.4 %, 32.7 %, 13.5 %, 6.6 %, 0.3 %, and 0.3 % positive effects on increasing HI among children. Also, BW has a 13.1 % decrease in HI among adults. So, decreasing the concentration of heavy metals can be considered the best action to decrease HI. Decreasing the cucumber intake rate is not a logical action because cucumber (*Cucumis sativus L.*), as a fruit, has many fibers and nutrients that are helpful for the body's function.

#### 3.2.2 Carcinoenic effect

**Fig. 4:**
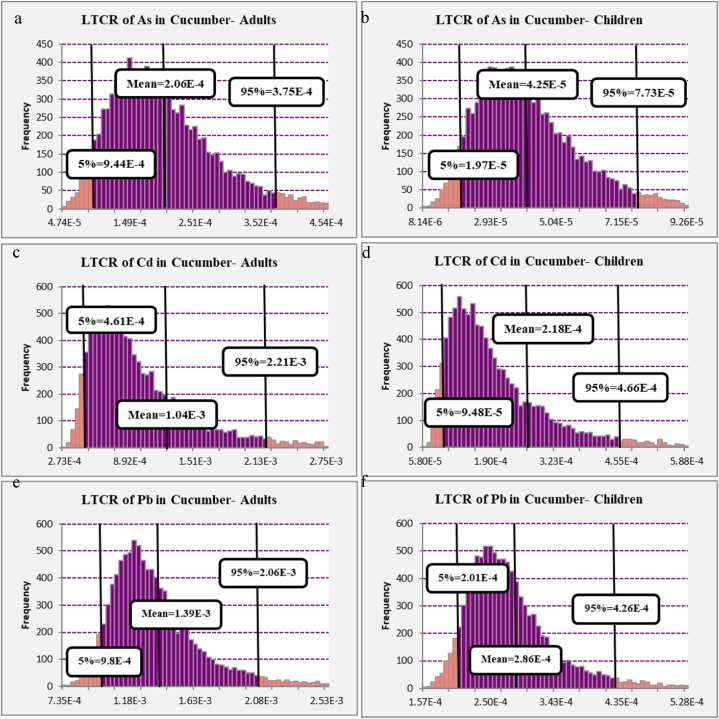
Histograms of the simulated ELCR of heavy metals in cucumber (*Cucumis sativus L.*) among children (b,d and f) and adults (a,c, and e).

The LTCR of heavy metals intake among children and adults that consume cucumber (Cucumis sativus L.) consumption is shown in Fig. 4. The carcinogenic effects of As, Cd, and Pb among adults are presented in Fig. 4 (a,c, and e). The 95th percentile of LTCR that related to As, Cd, and Pb among adults were 3.75E-04, 2.21E-03, and 2.06E-03, respectively. The 95th percentile of LTCR of As among adults was in the target range of WHO (1E-04 to 1E-06), so future and more investigation are needed before designating low priority or taking action. Also, the 95th percentile of LTCR related to Cd and Pb is about 1E-03, so more investigation is needed in the future before taking any action. The carcinogenic effects of As, Cd, and Pb among children are presented in Fig. 4 (b,d, and f). The 95th percentile of LTCR that related to As, Cd, and Pb among children was 7.73E-05, 4.66E-04, and 4.26E-04, respectively. According to these results, the 95th percentile of LTCR of As, Cd, and Pb among children was within the target range of WHO (1E-04 to 1E-06), so future and more investigation are needed before designating low priority or taking action. Also, the 95th percentile of LTCR related to Cd and Pb is about 1E-03, so more investigation is needed in the future before taking any action. The results showed that the carcinogenic risks of heavy metal intake via cucumber (*Cucumis sativus L.*) consumption among adults were higher than those among children, because the exposure duration of children (6 years) was lower than that of adults (70 years). Also, the cucumber (*Cucumis sativus L.*) intake rate of adults was higher than that of children, which is contrary to the results of Su et al. (2023) [70].

#### 3.2.3 ELCR and sensitivity analysis of heavy metals in cucumber (Cucumis sativus L.)

**Fig. 5:**
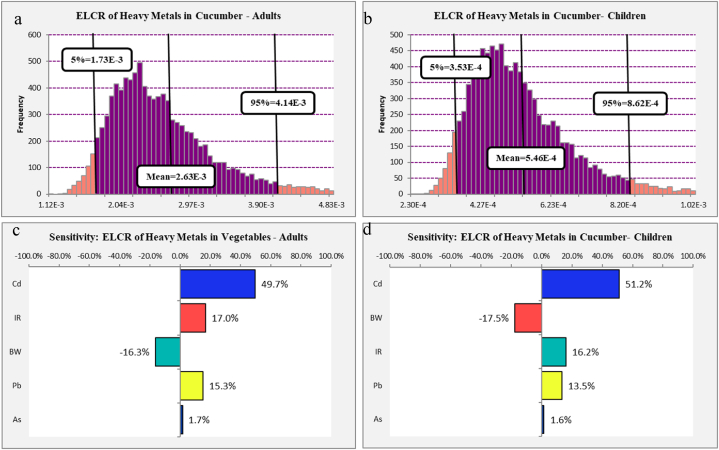
Histograms of the simulated ELCR (a,b) of heavy metals in cucumber (*Cucumis sativus L.*) among children and adults.

As mentioned above, heavy metals have an accumulation property in human tissues. So the accumulative and synergistic effects of increasing the carcinogenic risk over the long term should be considered. According to Eq. (6), the ELCRs were calculated, and results are presented in Fig. 5 a and b. These results present that the 95th percentile of ELCR of heavy metal intake via cucumber (Cucumis sativus L.) consumption among adults and children was 4.14E-03 and 8.62E-04, respectively; so for adults, more investigation is needed in the future before taking any action, and for children, the 95th percentile of ELCR was within the target range of WHO (1E-04 to 1E-06), so future and more investigation are needed before designating low priority or taking action.

The sensitivity analysis can help to determine the effect of each parameter on increasing or decreasing of the ELCR. The sensitivity analysis of calculated ELCR of heavy metals in cucumber (*Cucumis sativus L.*) among adults and children is presented in Fig. 5 c and **d**. The results show that for the ELCR of adults Cd, IR, Pb, and As have 49.7 %, 17 %, 15.3 %, and 1.7 % positive effects on increasing of ELCR among adults. Also, BW has a 16.3 % decreasing effect on ELCR among adults. The results of ELCR calculation for children shown that Cd, IR, Pb, and As have 51.2 %, 16.2 %, 13.5 %, and 1.6 % positive effects on increasing of ELCR among children. Also, BW has a 17.5 % decreasing effect on ELCR among children. So,for both group, decreasing the concentration of Cd is helpful to decrease the ELCR because about 50 % of calculated ELCR in children and adult related to Cd concentration.

## 4 Conclusion

The present study examined heavy metal (As, Pb, Cd, Cu, and Zn) concentrations and health concerns in cucumber (*Cucumis sativus L.*). Mean (±SD) concentrations of As, Pb, Cd, Cu, and Zn levels in cucumber (*Cucumis sativus L.*) samples were 157.87 ± 128.54, 33.81 ± 6.27, 288.46 ± 114.59, 35.22 ± 18.67, and 33.6 ± 18.1 μg/kg, respectively, which were lower than FAO/WHO standard. The 95th percentile of HI related to heavy metal intake via cucumber (*Cucumis sativus L.*) among children and adults were 2.64 and 1.75, respectively. Also, the 95th percentile of ELCR related to heavy metal were 8.26E-4 and 4.14E-3 among children and adults, respectively. Although As and Zn in cucumbers (*Cucumis sativus L.*) can harm children, reducing their amounts is the best way to obtain a lower HI. The 95th percentile of LTCR of As among adults and As, Cd, and Pb among children were in the WHO target range (1E-04 to 1E-06) so reducing the concentration of them can help to reduce overall LTCR. When HQ and LTCR are below the cut limits, reducing heavy metals in high-consumption meals is a good way to lower them. We can employ several strategies to reduce the consumption of heavy metals in cucumbers (*Cucumis sativus L.*). These include (i) conducting soil testing and remediation, (ii) managing water sources effectively, (iii) adopting organic farming practices that abstain from using synthetic fertilizers and pesticides, and (iv) washing and peeling. These can help remove surface contaminants, including heavy metals, before consumption. Despite the possibility of incomplete contamination removal, these methods can significantly reduce exposure levels. This study's limitations included a small sample size due to the high cost of measurement and a lack of variety in sampling locations. Also, if all markets in Iran were sampled in this study and the soil and water samples used for irrigation were also analyzed, we could more confidently generalize it to the studied Iranian population, which is a suggestion for future studies.

## Ethical approval

Not applicable.

## CRediT authorship contribution statement

**Mohammad Rezvani Ghalhari:** Writing – review & editing, Writing – original draft, Visualization, Validation, Software, Methodology, Formal analysis, Data curation, Conceptualization. **Nayereh Rezaei Rahimi:** Writing – original draft, Validation, Investigation, Formal analysis, Data curation. **Mohammad Fahiminia:** Writing – review & editing, Writing – original draft, Supervision, Project administration, Conceptualization. **Elahe Noruzzade:** Writing – review & editing, Writing – original draft, Formal analysis, Data curation, Conceptualization. **Abooalfazl Azhdarpoor:** Writing – review & editing, Data curation, Conceptualization. **Zeynab Koochakzadeh:** Validation, Investigation, Data curation, Conceptualization. **Habib Vakili:** Visualization, Validation, Data curation. **Reza Fouladi-Fard:** Writing – review & editing, Writing – original draft, Visualization, Validation, Supervision, Resources, Project administration, Methodology, Funding acquisition, Formal analysis, Data curation, Conceptualization.

## Declaration of competing interest

The authors declare that they have no known competing financial interests or personal relationships that could have appeared to influence the work reported in this paper
